# The impact of diabetes, anemia, and renal function in the relationship between osteoporosis and fasting blood glucose among Taiwanese women: a cross-sectional study

**DOI:** 10.1186/s12905-023-02851-w

**Published:** 2024-01-03

**Authors:** Tzyy-Ling Chuang, Malcolm Koo, Yuh-Feng Wang

**Affiliations:** 1 Department of Nuclear Medicine, Dalin Tzu Chi Hospital, Buddhist Tzu Chi Medical Foundation, Chiayi, Taiwan; 2 School of Medicine, Tzu Chi University, Hualien, Taiwan; 3 Department of Nursing, Tzu Chi University of Science and Technology, Hualien, 970302 Taiwan; 4 Department of Nuclear Medicine, Taipei Veterans General Hospital, No.201, Sec. 2, Shipai Road, Beitou District, Taipei City, 11217 Taiwan; 5 Department of Biomedical Imaging and Radiological Sciences, National Yang Ming Chiao Tung University, Taipei, Taiwan; 6 Department of Medical Imaging and Radiological Technology, Yuanpei University of Medical Technology, Hsinchu, Taiwan

**Keywords:** anemia, Bone mineral density, Diabetes Mellitus, Estimated glomerular filtration rate, Osteoporosis

## Abstract

**Background:**

The aim of this study was to investigate the association between fasting blood glucose and osteoporosis in women with diabetes, anemia, and renal function.

**Methods:**

The medical records of women who underwent a general health examination at a regional hospital in southern Taiwan were retrospectively reviewed. Logistic regression analysis was performed to assess the association between osteoporosis and fasting blood glucose separately for the eight subgroups (diabetes or non-diabetes, anemia or non-anemia, normal or decreased renal function), adjusting for other clinical characteristics and laboratory findings.

**Results:**

A total of 11,872 women were included in the study. Among women with diabetes, anemia, and decreased renal function, an increment of 10 mg/dL in fasting blood glucose was associated with an increased risk of osteoporosis (adjusted odds ratio [aOR] = 1.57, *p* = 0.004). Among women without diabetes, fasting blood glucose was significantly associated with an increased risk of osteoporosis in those with anemia and normal renal function (OR = 1.14, *p* = 0.023) and those without anemia and normal renal function (OR = 1.04, *p* = 0.015), but these associations were not significant after adjusting for other covariates.

**Conclusions:**

Higher fasting blood glucose levels in women with diabetes, anemia, and decreased renal function were associated with an increased risk of osteoporosis. Clinicians should be vigilant about glucose control in patients with diabetes to reduce the risk of fracture.

## Introduction

Diabetes and osteoporosis are prevalent diseases with significant associated morbidity and mortality worldwide [1]. Well-known complications of diabetes include microvascular disease (nephropathy, retinopathy, and neuropathy), macrovascular disease (acute coronary syndrome), anemia, and stroke [2]. Both type 1 and 2 diabetes are associated with inferior bone quality and strength, leading to an increased fracture risk [3–5].

Diabetic kidney disease affects approximately 40% of people with diabetes and is the leading cause of chronic kidney disease worldwide [6]. It is also one of the main causes of death in these patients [7]. The cross-sectional association between renal function and bone mineral density (BMD) is strongest at higher stages of chronic kidney disease [8]. Despite decreased BMD in type 1 diabetes, BMD in type 2 diabetes is often normal or even slightly elevated compared with age-matched controls [9]. However, a cohort study of 1690 men and 1641 women in Taiwan found that type 2 diabetes was significantly associated with an increased risk of osteoporosis, especially among younger participants [10].

Patients with poorly controlled diabetes and those with diabetes and renal insufficiency are more likely to develop anemia [11]. A retrospective observational study of 19,059 adult patients with type 2 diabetes showed a higher prevalence of anemia (38.5%) in women with diabetes [12]. In another cross-sectional study of 249 patients with type 2 diabetes, poor glycemic control, decreased estimated glomerular filtration rate (eGFR), presence of diabetes-related complications, duration of diabetes > 10 years, and age > 60 years were significantly associated with the development of anemia in these patients [13]. Low hemoglobin levels are also associated with a high risk of osteoporosis in adults [14].

Given the existing ambiguity surrounding the relationship between blood glucose levels and osteoporosis in women, particularly those with coexisting conditions with diabetes, there is a need for more comprehensive research. Understanding how these conditions affect bone metabolism is crucial, as this knowledge can aid clinicians in effectively managing patients with diabetes, given the vital role of bone health in overall patient quality of life. Therefore, the present study aimed to explore the association between elevated fasting blood glucose levels and the risk of osteoporosis in women, focusing on the associations in those having various combinations of diabetes, anemia, and renal insufficiency.

## Materials and methods

### Study participants and variables

In this retrospective study, medical records of women who had undergone a general health examination at a regional teaching hospital in southern Taiwan between June 2014 and July 2020 were identified and reviewed. The study protocol was approved by the institutional review board of Dalin Tzu Chi Hospital (IRB No. B11001010), and the requirement for obtaining informed consent from patients was waived.

The medical records of the participants were reviewed to obtain information on the following: (1) anthropometric characteristics, including age and body mass index; (2) comorbidities, including hypertension, diabetes mellitus, hyperlipidemia, and chronic kidney disease; (4) laboratory findings, including hemoglobin, systolic blood pressure, low-density lipoprotein cholesterol (LDL-C), fasting blood glucose, estimated glomerular filtration rate (eGFR), and alkaline phosphatase (ALP). Participants with metal materials in the measured areas of bone mineral density were excluded from the study. In addition, those who smoked or consumed alcohol were excluded to eliminate the potential confounding effects of these risk factors for osteoporosis.

### Measurements

BMD at the lumbar spine and bilateral hips, including the total and femoral neck regions, was determined using dual-energy X-ray absorptiometry (DXA) with a DiscoveryWi DXA system (Hologic Inc., Marlborough, MA, USA). Participants were categorized into those with osteoporosis (T-score ≤ − 2.5 standard deviation [SD]) or non-osteoporosis (T-score > − 2.5 SD) based on the World Health Organization classification, as determined by their lowest T-score among the three measured sites [15].

### Subgroup categorization

Subgroups for stratified analysis were created based on information obtained from questionnaires and medical records. In accordance with the World Health Organization recommendations [16], anemia was defined as a hemoglobin level < 12.0 g/dL in women. Anemia levels were also classified as mild (hemoglobin level 11.0–11.9 g/dL), moderate (hemoglobin level 8.0–10.9 g/dL), and severe (hemoglobin level < 8.0 g/dL) in non-pregnant women [17].

Kidney function was evaluated by eGFR, which was calculated using the abbreviated Modification of Diet in Renal Disease (MDRD) equation [18]: 186 × (serum creatinine in mg/dL)^−1.154^ × (age)^−0.203^ × 0.742 (if female). Participants were divided into two groups based on their eGFR: decreased renal function if eGFR < 90 mL/min/1.73m^2^ and normal renal function if eGFR ≥ 90 mL/min/1.73m^2^. Eight subgroups were created based on the three factors of diabetes, anemia, and renal function. These subgroups were then analyzed separately in the regression analyses.

### Statistical analysis

Summary statistics were presented as means with SDs or as numbers with percentages, as appropriate. Differences in means or frequencies of characteristics and variables between the eight subgroups were evaluated using the Chi-square test or the independent t-test, as applicable.

Univariate logistic regression analysis was conducted using the osteoporotic state (osteoporosis or non-osteoporosis) as the dependent variable and fasting blood glucose, as well as other clinical characteristics and laboratory findings, as independent variables. Due to the presence of interactions between diabetes and anemia as well as anemia and renal function, stratified analyses were conducted based on the eight defined groups, which combined the statuses of diabetes, anemia, and renal impairment. Variables exhibiting a *p*-value less than 0.20 in the univariate analysis were subsequently included in the multiple logistic regression analysis to further assess their independent associations with osteoporosis. In addition, multiple logistic regression analyses were performed to explore the independent association between fasting blood glucose and osteoporosis, adjusting for clinical and laboratory variables that were significantly associated with osteoporosis in the univariate analysis.

All statistical analyses were performed using PASW Statistics for Windows, Version 18.0 (SPSS Inc., Chicago, IL, USA). A *p*-value of less than 0.05 was considered to indicate statistical significance.

## Results

### Characteristics of the study participants

**Fig. 1:**
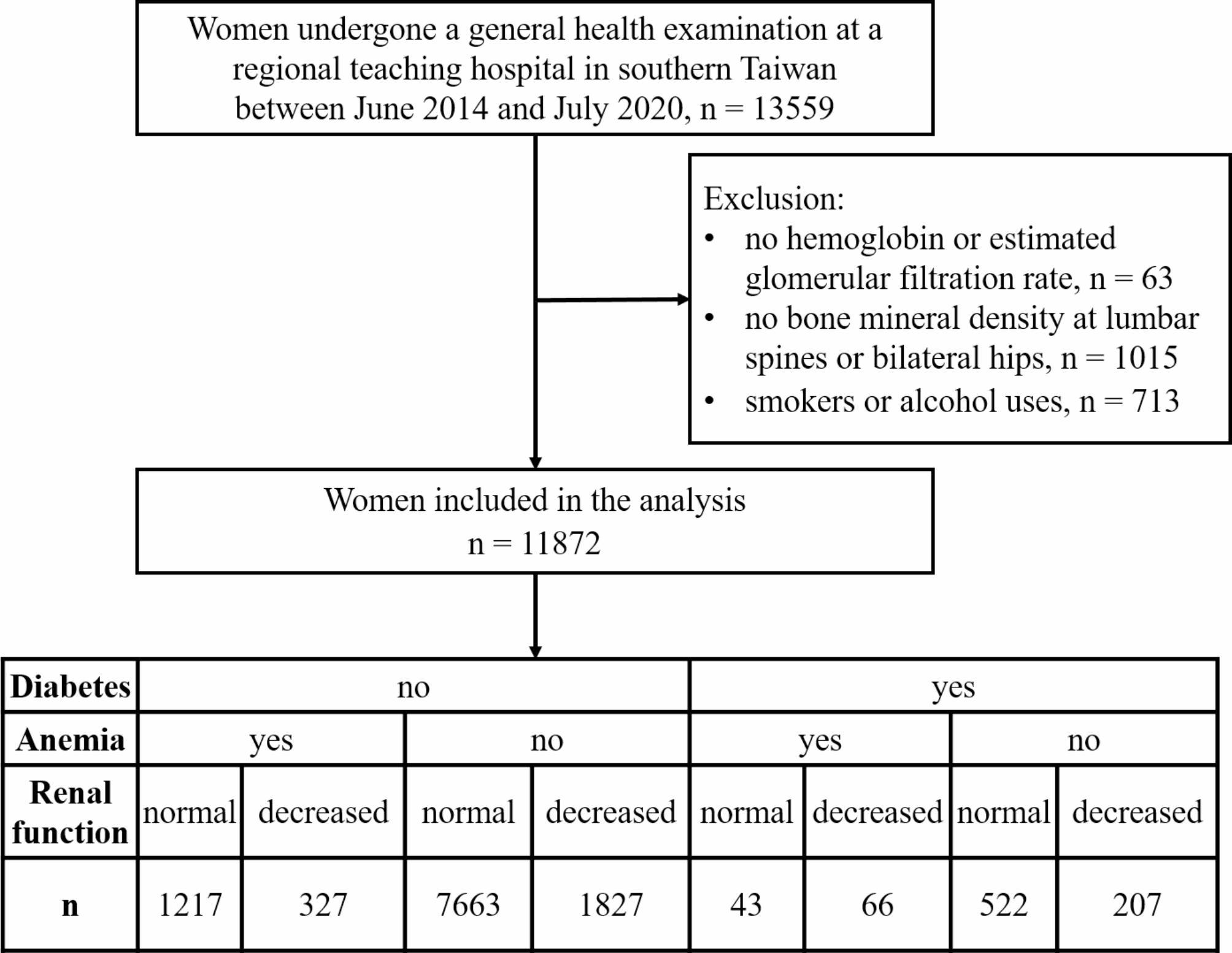
Study flowchart

A total of 11,872 women were included in the study, comprising 838 with diabetes, 11,034 without diabetes, 1,653 with anemia, 10,219 without anemia, 2,427 with an eGFR < 90 mL/min/1.73 m², and 9,445 with an eGFR ≥ 90 mL/min/1.73 m². According to their T-score for BMD, 3312 of the women were classified as having osteoporosis, representing 27.9% of the study participants. Figure 1 presents a flowchart of the study participants.

Table 1 shows the demographic and clinical characteristics of women, stratified by renal function, diabetes, and anemia status. First, women with normal renal function and anemia who also had diabetes were significantly older, had higher body mass index, higher proportions of hypertension and hyperlipidemia, higher systolic blood pressure, lower LDL-C, higher fasting blood glucose, and higher proportions of osteoporosis, compared to those without diabetes. Second, women with normal renal function but without anemia who had diabetes were significantly older, had higher body mass index, higher hemoglobin, higher proportions of hypertension and hyperlipidemia, higher systolic blood pressure, a lower LDL-C, and higher fasting blood glucose, compared to those without diabetes. Third, women with decreased renal function and anemia who had diabetes were significantly older, had higher body mass index, higher proportions of hypertension and hyperlipidemia, higher systolic blood pressure, lower LDL-C, higher fasting blood glucose, and lower eGFR, compared to those without diabetes. Finally, women with decreased renal function and without anemia who had diabetes were significantly older, had higher body mass index, lower hemoglobin, higher proportions of hypertension and hyperlipidemia, lower LDL-C, higher fasting blood glucose, and lower eGFR, compared to those without diabetes. In addition, the level of anemia was not significant different between diabetes and non-diabetes in normal renal function and decreased renal function.

**Table 1:** Demographic and clinical characteristics of women (*N* = 11,872)

Variable	eGFR ≥ 90 (normal)			eGFR < 90 (decreased renal function)		
	Diabetes	Non-diabetes	*p*	Diabetes	Non-diabetes	*p*
***Anemia***						
*N* (%)	43 (3.4)	1217 (96.6)		66 (16.8)	327 (83.2)	
Age (years)	58.7 (10.8)	50.8 (11.0)	< 0.001	67.5 (8.8)	62.3 (11.4)	< 0.001
Body mass index (kg/m^2^)	26.0 (5.6)	22.8 (3.3)	0.001	26.3 (4.7)	23.8 (3.4)	< 0.001
Hemoglobin (g/dL)	11.0 (1.1)	10.8 (1.2)	0.192	11.2 (0.7)	11.1 (0.9)	0.565
Anemia level, *n* (%)			0.383			0.764
Mild	29 (67.4)	694 (57.0)		47 (71.2)	224 (68.5)	
Moderate	13 (30.2)	472 (38.8)		19 (28.8)	101 (30.9)	
Severe	1 (2.3)	51 (4.2)		0	2 (0.6)	
Hypertension, *n* (%)	19 (44.2)	101 (8.3)	< 0.001	46 (69.7)	104 (31.8)	< 0.001
Hyperlipidemia, *n* (%)	8 (18.6)	30 (2.5)	< 0.001	24 (36.4)	22 (6.7)	< 0.001
Chronic kidney disease, *n* (%)	0 (0.0)	5 (0.4)	> 0.999	9 (13.6)	25 (7.6)	0.114
Systolic blood pressure (mmHg)	131.2 (18.3)	119.5 (19.7)	< 0.001	135.4 (22.6)	126.3 (21.6)	0.002
Low-density lipoprotein cholesterol (mg/dL)	93.2 (25.2)	104.8 (27.1)	0.005	88.4 (26.5)	111.3 (29.2)	< 0.001
Fasting blood glucose (mg/dL)	148.4 (44.5)	96.7 (11.7)	< 0.001	139.8 (40.1)	99.0 (10.6)	< 0.001
eGFR (mL/min/1.73m^2^)	118.3 (26.5)	120.3 (22.0)	0.558	60.7 (20.4)	72.2 (18.4)	< 0.001
Alkaline phosphatase (IU/L)	70.4 (19.5)	65.0 (28.4)	0.215	68.4 (21.2)	71.3 (24.4)	0.367
Osteoporosis, *n* (%)	13 (30.2)	223 (18.3)	0.049	27 (40.9)	136 (41.6)	0.918
***Non-anemia***						
*N* (%)	522 (6.4)	7663 (93.6)		207 (10.2)	1827 (89.8)	
Age (years)	62.0 (8.2)	55.5 (10.5)	< 0.001	65.3 (7.9)	61.6 (9.6)	< 0.001
Body mass index (kg/m^2^)	25.2 (4.0)	23.4 (3.4)	< 0.001	25.4 (3.4)	24.4 (3.6)	< 0.001
Hemoglobin (g/dL)	13.6 (0.9)	13.5 (0.8)	0.002	13.3 (0.9)	13.4 (0.9)	0.018
Hypertension, *n* (%)	236 (45.2)	1110 (14.5)	< 0.001	114 (55.1)	532 (29.1)	< 0.001
Hyperlipidemia, *n* (%)	144 (27.6)	392 (5.1)	< 0.001	78 (37.7)	148 (8.1)	< 0.001
Chronic kidney disease, *n* (%)	4 (0.8)	39 (0.5)	0.351	7 (3.4)	29 (1.6)	0.086
Systolic blood pressure (mmHg)	131.3 (19.3)	124.1 (20.5)	< 0.001	131.2 (19.5)	129.9 (21.5)	0.371
Low-density lipoprotein cholesterol (mg/dL)	105.6 (30.7)	119.6 (30.9)	< 0.001	99.0 (30.3)	123.9 (31.7)	< 0.001
Fasting blood glucose (mg/dL)	150.0 (43.1)	100.7 (15.3)	< 0.001	149.8 (43.7)	102.3 (15.1)	< 0.001
eGFR (mL/min/1.73m^2^)	116.6 (20.8)	116.8 (19.4)	0.795	75.2 (11.9)	79.6 (9.0)	< 0.001
Alkaline phosphatase (IU/L)	76.4 (22.8)	77.1 (42.3)	0.730	74.8 (22.5)	77.9 (23.1)	0.073
Osteoporosis, *n* (%)	146 (28.0)	2097 (27.4)	0.765	58 (28.0)	612 (33.5)	0.112

eGFR: estimated glomerular filtration rate

Numbers in the table are mean (standard deviation) unless otherwise specified

Table 2 presents the BMD and T-score of women, stratified by renal function, diabetes, and anemia. In women with anemia, BMD and T-scores at the three measured sites showed no significant differences between those with and without diabetes, regardless of renal function status. Conversely, in women without anemia, the bilateral hip total BMD and T-scores were higher in those with diabetes, irrespective of renal function. In addition, women with decreased renal function, diabetes also had significantly higher lumbar spine BMD and T-score, as well as right hip femoral neck BMD, and left hip femoral neck BMD and T-score.

**Table 2:** Bone mineral density and T-score of women (*N* = 11,872)

Variable	eGFR ≥ 90 (normal)			eGFR < 90 (decreased renal function)		
	Diabetes	Non-diabetes	*p*	Diabetes	Non-diabetes	*p*
***Anemia***						
*N* (%)	43 (3.4)	1217 (96.6)		66 (16.8)	327 (83.2)	
Lumbar spine BMD (g/cm^2^)	0.935 (0.182)	0.929 (0.156)	0.814	0.870 (0.163)	0.854 (0.174)	0.493
Lumbar spine T-score	−0.6 (1.6)	−0.7 (1.4)	0.853	−1.1 (1.4)	−1.3 (1.5)	0.431
Right hip femoral neck BMD (g/cm^2^)	0.667 (0.149)	0.678 (0.123)	0.569	0.613 (0.134)	0.610 (0.125)	0.864
Right hip femoral neck T-score	−1.3 (1.3)	−1.2 (1.1)	0.428	−1.8 (1.3)	−1.8 (1.2)	0.783
Right hip total BMD (g/cm^2^)	0.827 (0.139)	0.812 (0.127)	0.459	0.786 (0.137)	0.763 (0.136)	0.220
Right hip total T-score	−0.2 (1.2)	−0.3 (1.1)	0.426	−0.5 (1.2)	−0.7 (1.2)	0.226
Left hip neck femoral BMD (g/cm^2^)	0.671 (0.144)	0.686 (0.125)	0.430	0.627 (0.135)	0.618 (0.125)	0.624
Left hip neck femoral T-score	−1.3 (1.4)	−1.1 (1.2)	0.433	−1.7 (1.3)	−1.7 (1.2)	0.661
Left hip total BMD (g/cm^2^)	0.785 (0.143)	0.781 (0.130)	0.842	0.753 (0.137)	0.732 (0.137)	0.247
Left hip total T-score	−0.6 (1.2)	−0.6 (1.1)	0.772	−0.8 (1.2)	−1.0 (1.2)	0.258
***Non-anemia***						
*N* (%)	522 (6.4)	7663 (93.6)		207 (10.2)	1827 (89.8)	
Lumbar spine BMD (g/cm^2^)	0.876 (0.147)	0.881 (0.153)	0.431	0.894 (0.138)	0.856 (0.149)	< 0.001
Lumbar spine T-score	−1.1 (1.3)	−1.1 (1.3)	0.593	−0.9 (1.2)	−1.3 (1.3)	< 0.001
Right hip femoral neck BMD (g/cm^2^)	0.646 (0.119)	0.645 (0.112)	0.888	0.639 (0.110)	0.622 (0.110)	0.035
Right hip femoral neck T-score	−1.5 (1.1)	−1.5 (1.1)	0.799	−1.6 (1.0)	−1.7 (1.0)	0.051
Right hip total BMD (g/cm^2^)	0.795 (0.135)	0.782 (0.122)	0.039	0.801 (0.120)	0.778 (0.121)	0.010
Right hip total T-score	−0.5 (1.1)	−0.6 (1.1)	0.007	−0.4 (1.0)	−0.6 (1.0)	0.015
Left hip femoral neck BMD (g/cm^2^)	0.653 (0.117)	0.654 (0.112)	0.766	0.649 (0.109)	0.631 (0.112)	0.030
Left hip femoral neck T-score	−1.4 (1.1)	−1.4 (1.1)	0.894	−1.4 (1.1)	−1.6 (1.1)	0.021
Left hip total BMD (g/cm^2^)	0.776 (0.133)	0.760 (0.123)	0.007	0.785 (0.114)	0.754 (0.123)	0.001
Left hip total T-score	−0.6 (1.1)	−0.8 (1.1)	0.001	−0.6 (1.0)	−0.8 (1.1)	< 0.001

BMD: bone mineral density

Numbers in the table are mean (standard deviation) unless otherwise specified

### Univariate logistic regression analysis of osteoporosis

Among women without diabetes, 3068 of them (27.8%) were diagnosed with osteoporosis. Logistic regression analysis (Table 3) showed that several factors were significantly associated with an increased risk of osteoporosis in this group, including older age, lower body mass index, higher hemoglobin, non-anemia, hypertension, hyperlipidemia, higher systolic blood pressure, higher LDL-C, higher fasting blood glucose, lower eGFR, and higher ALP levels.

**Table 3:** Univariate logistic regression analysis of factors associated with osteoporosis in women without diabetes (*N* = 11,034)

Variable	Odds ratios (95% confidence interval)	*p*
Age (/10 years)	2.74 (2.60‒2.89)	< 0.001
Body mass index (kg/m^2^)	0.89 (0.88‒0.90)	< 0.001
Hemoglobin (g/dL)	1.05 (1.02‒1.09)	0.002
Anemia	0.76 (0.67‒0.86)	< 0.001
Hypertension	1.59 (1.43‒1.77)	< 0.001
Hyperlipidemia	1.36 (1.14‒1.62)	0.001
Chronic kidney disease	1.38 (0.91‒2.10)	0.128
Systolic blood pressure (/10 mmHg)	1.09 (1.07‒1.11)	< 0.001
Low-density lipoprotein cholesterol(/10 mg/dL)	1.02 (1.01‒1.04)	0.001
Fasting blood glucose (/10 mg/dL)	1.04 (1.01‒1.07)	0.005
eGFR (/10 mL/min/1.73m^2^)	0.92 (0.90‒0.93)	< 0.001
Alkaline phosphatase (/10 mg/dL)	1.22 (1.19‒1.24)	< 0.001

eGFR: estimated glomerular filtration rate

Among women with diabetes, 244 (29.1%) were diagnosed with osteoporosis. The results of the logistic regression analysis (Table 4) revealed that increased risk of osteoporosis was significantly associated with older age, lower body mass index, lower hemoglobin, and higher ALP levels.

**Table 4:** Univariate logistic regression analysis of factors associated with osteoporosis in women with diabetes (*N* = 838)

Variable	Odds ratios (95% confidence interval)	*p*
Age (/10 years)	2.57 (2.07‒3.20)	< 0.001
Body mass index (kg/m^2^)	0.86 (0.82‒0.90)	< 0.001
Hemoglobin (g/dL)	0.83 (0.73‒0.93)	0.002
Anemia	1.49 (0.98‒2.28)	0.063
Hypertension	0.98 (0.73‒1.32)	0.899
Hyperlipidemia	1.03 (0.75‒1.42)	0.863
Chronic kidney disease	1.64 (0.66‒4.07)	0.283
Systolic blood pressure (/10 mmHg)	0.98 (0.91‒1.06)	0.688
Low-density lipoprotein cholesterol (/10 mg/dL)	0.99 (0.94‒1.04)	0.655
Fasting blood glucose (/10 mg/dL)	0.98 (0.95‒1.02)	0.309
eGFR (/10 mL/min/1.73m^2^)	0.96 (0.91‒1.01)	0.117
Alkaline phosphatase (/10 mg/dL)	1.13 (1.06‒1.21)	< 0.001

eGFR: estimated glomerular filtration rate

Table 5 shows the results of univariate logistic regression analysis assessing the association between fasting blood glucose and osteoporosis, stratified by diabetes, anemia, and renal function status. In women without diabetes and with normal renal function, an increase of 10 mg/dL in fasting blood glucose was significantly associated with an increased risk of osteoporosis in those with anemia (OR = 1.14, *p* = 0.023) and those without anemia (OR = 1.04, *p* = 0.015). Among women with diabetes, an increase of 10 mg/dL in fasting blood glucose was significantly associated with an increased risk of osteoporosis only in those with anemia and decreased renal function (OR = 1.25, *p* = 0.010).

**Table 5:** Univariate logistic regression analysis of the association between osteoporosis and fasting blood glucose in women stratified by diabetes, anemia, and renal function

Disease status			*n*	odds ratios (95% confidence interval)	*p*
Diabetes	Anemia	Renal function			
non-diabetes	anemia	normal	1217	1.14 (1.02‒1.27)	0.023
non-diabetes	anemia	decreased	327	1.12 (0.91‒1.38)	0.285
non-diabetes	non-anemia	normal	7663	1.04 (1.01‒1.07)	0.015
non-diabetes	non-anemia	decreased	1827	0.94 (0.88‒1.01)	0.084
Diabetes	anemia	normal	43	0.87 (0.72‒1.05)	0.136
diabetes	anemia	decreased	66	1.25 (1.05‒1.47)	0.010
diabetes	non-anemia	normal	522	0.97 (0.92‒1.02)	0.180
diabetes	non-anemia	decreased	207	0.98 (0.91‒1.05)	0.579

### Multiple logistic regression analysis of osteoporosis and fasting blood glucose

Table 6 presents the results of the multiple logistic regression analysis after adjusting for age, body mass index, hemoglobin, systolic blood pressure, LDL-C, eGFR, and ALP. Among women with diabetes, an increase of 10 mg/dL in fasting blood glucose was independently and significantly associated with an increased risk of osteoporosis in those with concurrent anemia and decreased renal function (adjusted OR = 1.57, *p* = 0.004). Conversely, for women without diabetes, fasting blood glucose was not significantly associated with osteoporosis after adjusting for other covariates, irrespective of their anemia or renal function status.

**Table 6:** Multiple logistic regression analysis of the association between osteoporosis and fasting blood glucose in women stratified by diabetes, anemia, and renal function

Disease status			*n*	odds ratios (95% confidence interval)	*p*
Diabetes	Anemia	Renal function			
non-diabetes	anemia	normal	1217	1.01 (0.88‒1.17)	0.847
non-diabetes	anemia	decreased	327	0.91 (0.67‒1.22)	0.513
non-diabetes	non-anemia	normal	7663	0.98 (0.94‒1.02)	0.289
non-diabetes	non-anemia	decreased	1827	0.93 (0.86‒1.02)	0.114
diabetes	anemia	normal	43	0.92 (0.62‒1.35)	0.656
diabetes	anemia	decreased	66	1.57 (1.16‒2.14)	0.004
diabetes	non-anemia	normal	522	0.95 (0.89‒1.00)	0.070
diabetes	non-anemia	decreased	207	0.97 (0.90‒1.05)	0.508

All models were adjusted for age, body mass index, hemoglobin, systolic blood pressure, low-density lipoprotein cholesterol, estimated glomerular filtration rate, and alkaline phosphatase

## Discussion

### Osteoporosis, bone mineral density, and fasting blood glucose

Osteoporosis is a silent disease that is associated with substantial morbidity, mortality, and economic burden [19]. Both type 1 and type 2 diabetes have been linked to an increased risk for osteoporosis-related fractures. However, the relationship between diabetes and BMD is still inconclusive. Table 7 presents studies that examine the relationships between diabetes, anemia, and fasting blood glucose levels in connection with osteoporosis, bone mineral density, and the risk of fractures. A systematic review of 47 articles published between January 1950 and October 2010 revealed that 26 articles showed increased BMD in patients with diabetes, 13 articles showed decreased BMD, and eight articles reported no difference in bone mass [20]. In patients with type 2 diabetes, factors such as obesity, increased bone load, and insulin resistance can contribute to hyperinsulinemia, leading to increased bone formation [21]. Yet, despite having higher BMD, these patients are still at an increased risk of non-vertebral fractures [22]. In this study, increased BMD was observed among women with diabetes and without anemia only in specific measured sites, regardless of renal function. Older age and female sex were associated with an increased risk of osteoporosis [23], and the association was also observed among those with type 2 diabetes [24]. Being overweight or obese and having a disease duration over five years have also been implicated in the development of osteoporosis. Uncontrolled blood glucose levels are a major contributor to osteoporosis [24].

**Table 7:** Comparisons of studies evaluating the association between predictor and outcome variable

Predictor	Outcome variable	Association	Study population	Definition of outcome variable	Reference
Diabetes	bone quality	−			[1]
Diabetes	fracture	+			[3–5, 22]
Diabetes	bone mineral density	+	review, 26 articles	comprehensive review of 47 studies	[20]
Diabetes	bone mineral density	NS	review, 8 articles		
Diabetes	bone mineral density	−	review, 13 articles		
Diabetes	body mass index	+			[21]
Diabetes	osteoporosis	+	*n* = 3331, Taiwan retrospective cohort study, ≥ 40 years from the 2002 TwSHHH linked to the NHIRD, over a median follow-up duration of 13.6 years	incident osteoporosis based on ICD9-CM codes or the use of anti-osteoporotic agents	[10]
Diabetes	osteoporosis	−	*n* = 96,626, Korean cohort study, over 55 years received health check-up in 2009 linked to NHIS, average follow-up period 4.14 person-years	incident osteoporosis basis of ICD-code with newly registered	[26]
Diabetes	anemia	+			[12]
Diabetes (older males)	osteoporosis	−	*n* = 553, Iran cross-sectional study, ≥ 60 years from Amirkola health and ageing project in 2011–2012	osteoporosis based on T-score ≤ − 2.5 at either lumbar spine or femoral neck, bone mineral density by DXA	[68]
	anemia	−			
Iron deficiency anemia	osteoporosis	+	Iron deficiency anemia (*n* = 35,751) and no iron deficiency anemia (*n* = 178,755) sampled individuals from the 1998–2012 Taiwan NHIRD, cross-sectional study	osteoporosis based on ICD-9-CM code T score < 2.5 by DXA	[43]
Hemoglobin	osteoporosis	−	*n* = 139,520, osteoporosis and control 1:1 matched, Korean cross-sectional study, ≥ 40 years from NHIS Health Screening Cohort data, 2002–2013	osteoporosis based on ICD-10 codes	[14]
Hemoglobin & diabetes	osteoporosis (older men)	−	*n* = 573, China cross-sectional study, ≥ 60 years from Xuanwu Hospital, 2016/07–2018/05	osteoporosis based on T-score ≤ − 2.5 at any of lumbar spine, femoral neck and hip sites, bone mineral density by DXA	[72]
Fasting blood glucose + non-diabetes (women)	bone mineral density	+	*n* = 2367, China cross-sectional study, > 65 years, three medical examination centers, 2019/01–2020/12,	osteoporosis based on T-score ≤ − 2.5 at any of lumbar spine, femoral neck and hip sites, bone mineral density by DXA	[78]
	osteoporosis	−			
Fasting blood glucose	osteoporosis	+	n = 101, Vietnam type-2 diabetic patients, cross-sectional study, ≥ 30 years, admitted to 175 Military Hospital for treatment, 2018/04–2019/03	DXA	[24]
Fasting blood glucose or diabetes	bone mineral density	+	T2DM *n* = 1719, non-diabetes *n* = 6401, Taiwan cross-sectional study, 50–90 years from Health Examination Database of a hospital, 2010/01–2015/12	osteoporosis based on T-score ≤ − 2.5 at lumbar spine or femoral neck, bone mineral density by DXA	[21]
	osteoporosis	−			
Fasting blood glucose	osteoporosis, diabetes with high-increasing fasting blood glucose in men	+	*n* = 18,313, China, cohort study, physical examinations at a center during 2008–2018, more than 4-year follow-up period	incidence of osteoporosis based on T-score ≤ − 2.5 at lumbar spine, bone mineral density by DXA	[25]
	osteoporosis, non-diabetes with elevated-increasing fasting blood glucose in women	−			
Fasting blood glucose	vBMD	NS	*n* = 10,309, China health check-up cohort, 2018–2019	vBMD, lumbar spine by QCT	[88]
	aBMD (men)	+		aBMD, lumbar spine, by DXA	
Insulin resistance (HOMA-IR) + diabetes	osteoporosis (women)	+	*n* = 234, China, cross-sectional study, department of Endocrinology and Metabolism at a hospital, 2016/02–2018/08	osteoporosis based on T-score ≤ − 2.5 at lumbar spine, bone mineral density by DXA	[31]
Insulin resistance (HOMA-IR) + non-diabetes, postmenopausal women	aBMD	NS	*n* = 146, Study of Women’s Health Across the Nation, cross-sectional study	aBMD at lumbar spine and total hip by DXA	[89]
	vBMD and trabecular and cortical thickness	+		vBMD and bone microarchitecture by HR-pQCT, 2008/09–2011/04	

+: positive correlation; −: negative (inverse) correlation; NS: no significant correlation

aBMD: areal bone mineral density; DXA: Dual-energy X-ray Absorptiometry; HOMA-IR: Homeostatic Model Assessment of Insulin Resistance; HR-pQCT: High-Resolution Peripheral Quantitative Computed Tomography; ICD9-CM: International Classification of Diseases, Ninth Revision, Clinical Modification; NHIRD: National Health Insurance Research Database; TwSHHH: Taiwanese Survey on Prevalence of Hypertension, Hyperglycemia, and Hyperlipidemia; vBMD: volumetric bone mineral density

Some studies, but not all, have shown that higher fasting blood glucose was associated with increased BMD and a reduced risk of osteoporosis in patients both with and without diabetes [21, 25, 26]. Considering the temporal trajectories of fasting plasma glucose, the relationship between fasting blood glucose and osteoporosis risk appears bidirectional. In individuals with diabetes, those who maintained high levels of fasting blood glucose over time had the highest risk of osteoporosis (OR = 3.09; 95% [confidence interval] CI 1.16–8.22), and those with moderate-high fasting blood glucose levels had the second-highest risk (OR = 2.49; 95% CI 1.02–6.12) in men. Among those without diabetes, an elevated-increasing trajectory of fasting blood glucose was inversely associated with osteoporosis risk in women (OR = 0.62; 95% CI 0.43–0.88) [25].

### Diabetes and osteoporosis

Fasting blood glucose is a key indicator for diagnosing diabetes and evaluating glucose homeostasis. It is influenced by various cytokines secreted by bone and regulates the differentiation and maturation of osteoblasts [27]. Elevated blood glucose levels can stimulate collagen synthesis and bone formation by osteoblasts [28, 29]. However, a high glucose state with insulin resistance could impair bone formation, leading to diabetic bone disease [27, 30]. A higher degree of insulin resistance was associated with an increased risk of osteoporosis [31]. Several candidate loci affecting fasting glucose and BMD have been identified, such as rs6867040 on ITGA1 [32]. It is essential to consider not only the temporal trajectory of fasting blood glucose but also comorbidities in its influence on osteoporosis. To date, no studies have examined the potential impact of combining hemoglobin and eGFR, which are affected by diabetes, on the relationship between fasting blood glucose and osteoporosis.

The main finding of the current study was that fasting blood glucose was independently and significantly associated with an increased risk of osteoporosis in women with diabetes, anemia, and decreased renal function. Anemia and renal insufficiency, common complications of diabetes, can contribute to osteoporosis development. Elevated blood glucose levels may adversely impact bone matrix properties [33], directly or indirectly through sarcopenia [34]. Hyperglycemia and the accumulation of advanced glycation end products (AGEs) in collagen can lead to decreased bone mass and strength, increasing fracture risk [35, 36]. Persistent elevated blood glucose levels can result in the formation of AGEs, impairing osteoblast function and leading to osteoporosis [37]. Bone formation markers osteocalcin and amino-terminal propeptide of procollagen type 1 (PINP) were reduced in patients with diabetes [38, 39], and their levels were inversely correlated with glucose levels [40]. This finding supports the notion that patients with type 2 diabetes have lower biochemical indices of bone formation than controls. Recent reports indicated that patients with poor glycemic control had lower 25-hydroxy vitamin D levels [41], which may be a biological cause of osteoporosis.

### Anemia and osteoporosis

Anemia, particularly when caused by nutritional deficiencies, can compromise bone health due to inadequate nutrient supply necessary for bone formation [14]. Iron deficiency anemia, a common complication of chronic kidney disease [42], is an independent risk factor for osteoporosis [43]. Among patients with comorbidities, a consistent association between low hemoglobin levels and osteoporosis has been observed [14]. Chronic iron deficiency anemia is thought to induce bone resorption, increasing the risk of osteoporosis [44]. Reduced blood volume promotes the proliferation of hematopoietic cells, including osteoclasts, leading to increased bone resorption. While blood loss can stimulate osteoblast formation, heightened bone resorption may disrupt bone remodeling cycles, potentially inducing osteoblast fatigue [45]. In addition, inflammation may also mediate the association between anemia and osteoporosis. Studies have shown that pro-inflammatory cytokines can affect hematopoiesis [46], and interleukin 6 (IL-6) may promote osteoclast differentiation and activation [47]. Anemia, characterized by decreased red blood cells or hemoglobin concentration, leads to reduced oxygen delivery to tissues, including bone. Insufficient oxygen supply can negatively impact bone cells and remodeling [48]. Chronic hypoxia may increase oxidative stress levels, and acidification of the extracellular matrix can impair bone metabolism [49]. Hypoxia has been demonstrated to decrease BMD in both humans and rats [50]. The coexistence of chronic diseases could amplify the effect of an anemic condition on osteoporosis [49]. Hypoxia-inducible factors (HIFs), crucial in the cellular response to hypoxia [51], with HIF1-α and HIF2-α may impact osteoblast and osteoclast functions in bone homeostasis [52, 53]. Iron deficiency directly or indirectly impedes osteoblastic differentiation and promotes osteoclastic differentiation by leading to hypoxia-induced increases in HIF-1/2α expression and a reduction in active vitamin D levels [54]. Adequate nutrient intake and normal red blood cell production generally support bone health in non-anemic individuals, but factors such as hormonal balance and physical activity can also play significant roles.

### Renal function and osteoporosis

A Korean study supported the association between eGFR and BMD, suggesting that osteoporosis might be highly prevalent in patients with moderate to severe chronic kidney disease [55]. Long-term complications of diabetes, particularly micro- or macroalbuminuria and decreased renal function, have been found to be associated with osteoporosis [23]. Calcium, phosphate, vitamin D, and parathyroid hormone (PTH) are vital for bone formation and mineralization, maintaining normal bone homeostasis. This process relies on the interplay of various factors such as PTH, vitamin D, calcitonin, fibroblast growth factor-23, and klotho, which regulate calcium and phosphate levels. The parathyroid gland, kidney, and intestine are involved in this system [56]. Serum levels of vitamin D and PTH play pivotal roles as mediators in the link between renal function and bone health [8]. Reduced renal function can lead to decreased activation of vitamin D and retention of phosphorus, potentially lowering serum calcium levels and leading to increased PTH secretion, then secondary hyperparathyroidism. Elevated PTH levels can stimulate bone resorption, leading to bone loss and decreased bone mineral density, which can increase bone turnover and osteoporosis risk [57]. Metabolic acidosis, commonly seen in advanced chronic kidney disease, can lead to bone buffering, where bone minerals are used to buffer the excess acid, resulting in bone loss [58, 59]. With normal renal function, the body can effectively regulate mineral balance, thereby supporting optimal bone health, provided other risk factors for osteoporosis are controlled.

### Interactions between diabetes, anemia, and renal function

Diabetes is one of the leading causes of chronic kidney disease and can result in diabetic nephropathy, which is characterized by progressive kidney damage [60]. The decline in eGFR is nearly twice as rapid as in patients with type 2 diabetes mellitus compared to those without diabetes [61]. The interplay between diabetes and chronic kidney disease involves complex mechanisms, including inflammation, oxidative stress, and alterations in the renin-angiotensin-aldosterone system, all of which can accelerate renal dysfunction in patients with diabetes [62]. Diabetic nephropathy is associated with a higher risk of anemia in patients with diabetes. Moreover, the production of erythropoietin decreases as eGFR declines [63]. Chronic kidney disease can result in reduced erythropoietin production by the kidneys, leading to anemia due to inadequate stimulation of red blood cell production in the bone marrow [64]. In diabetes, chronic hyperglycemia and insulin resistance can impair erythropoietin production, contributing to anemia [13]. Approximately 90% of erythropoietin is produced by the kidneys [65]. With the decline of functional renal tissue in patients with chronic kidney disease, the body’s ability to produce sufficient erythropoietin in response to hypoxia in the kidney diminishes [66]. The prevalence of anemia gradually increases with the progressive stages of chronic kidney disease, being higher in patients with diabetes compared to non-diabetes counterparts, with diabetes being an independent predictor of anemia occurrence [67].

**Fig. 2:**
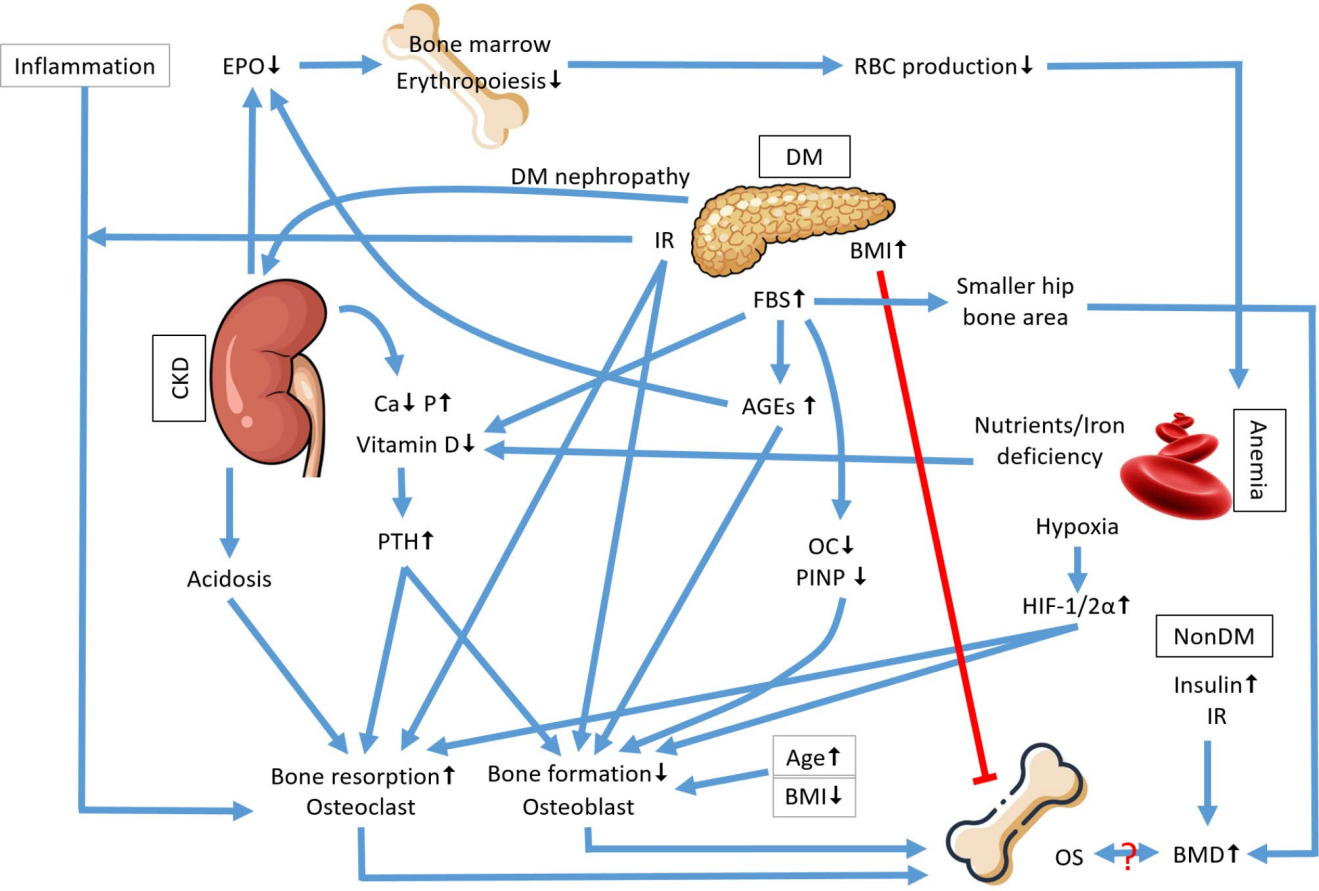
Schematic representation of biological interactions among diabetes, anemia, and renal function in the pathogenesis of osteoporosis. The diagram illustrates potential pathways through which diabetes mellitus (DM), anemia, and chronic kidney disease (CKD) may influence bone mineral density (BMD) and contribute to the development of osteoporosis (OS). Key molecular and physiological factors include advanced glycation end products (AGEs), erythropoietin (EPO), hypoxia-inducible factors (HIF), and parathyroid hormone (PTH), among others. The figure also denotes the roles of fasting blood glucose (FBS), insulin resistance (IR), and body mass index (BMI) in the context of bone metabolism, highlighting the complex interplay of systemic factors in bone health. (Ca: calcium; P: phosphorus; OC: osteocalcin; PINP: amino-terminal propeptide of type I procollagen)

### Multifactorial influences on osteoporosis

Diabetes, obesity, and higher muscle strength in older men were associated with a lower prevalence of osteoporosis and anemia [68]. Age, sex, body weight, body mass index, high-density lipoprotein cholesterol (HDL-C), and diabetes are key predictors of osteoporosis [69]. Moreover, plasma levels of LDL-C and HDL-C have opposite effects on BMD in males and females. [70, 71]. Obesity, a significant risk factor for type 2 diabetes, might offer some protection against osteoporosis due to the positive correlation between body mass index and BMD [24].

The impact of diabetes with varying renal functions and anemic statuses on osteoporosis in women has not been thoroughly studied. However, higher levels of hemoglobin have been shown to be protective against osteoporosis in older men with type 2 diabetes [72]. Although patients with diabetes have higher BMD, they are still at increased risk of fracture due to poor bone quality [1]. BMD measurements alone do not fully reflect bone fragility in diabetes, as deterioration in bone quality, rather than loss of bone mass, is a critical factor.

A meta-analysis suggested fasting blood glucose might be a causal risk factor for a reduced hip bone area and increased BMD [73]. Poor glycemic control in type 2 diabetes was associated with increased fracture risk, despite higher BMD and thicker femoral cortex in these individuals [74]. As low BMD is linked with a greater risk of falls, maintaining normal blood glucose levels is vital for preventing osteoporosis. The detrimental effects of chronic high blood glucose levels on bone health should be acknowledged alongside other well-recognized complications of diabetes [75].

Chronically high fasting blood glucose levels can damage osteoblasts by glycosylating hemoglobin. This damage, combined with hyperinsulinemia, leads to harmful deposits in bone collagen. This issue, along with reduced serum levels of insulin-like growth factor, hypercalciuria, inflammation, microangiopathy, and progressive renal failure in diabetes, leads to poor bone health [76, 77]. In non-diabetic older women, higher fasting blood glucose level was associated with BMD. Conversely, low fasting blood glucose may increase the risk of osteoporosis [78].

Both diabetics and non-diabetics can exhibit insulin resistance and elevated insulin levels. Physiological concentrations of insulin can inhibit osteoclast activity while promoting the proliferation of bone-forming osteoblasts, along with the synthesis of collagen, ALP production, and glucose absorption. Insulin deficiency was associated with reduced bone mineralized surface area, osteoid surface, the rate of mineral deposition, as well as osteoblast activity [79]. Studies in mouse models have shown that, in the absence of hyperglycemia, increased insulin levels and insulin resistance might lead to lower bone turnover and higher areal BMD [80].

An inverse relationship exists between insulin resistance and BMD, suggesting that in type 2 diabetes mellitus, insulin resistance might diminish the beneficial effects of insulin on bones [31]. Our results from multiple logistic regression analysis suggested that while normal fasting blood glucose levels are essential for overall health, their influence on bone health in non-diabetic individuals might not be as significant as in those with diabetes. This is especially true when other risk factors for osteoporosis, such as decreased renal function and anemia, are not present.

### Limitations and future directions

This study has several limitations that warrant mention. First, it is based on data from a relatively healthy population, comprised of individuals who participated in health examinations. This may affect the generalizability of our findings. Second, while menopausal status may affect the risk of bone fracture [81], we lacked information on the menopausal status of our participants. Nevertheless, the potential impact of menopause was indirectly adjusted by including age in all our multiple regression models. Third, our questionnaire did not include questions about gravidity or parity [82], history of thyroid surgery [83], or thyroid-related diseases [84]. As a result, we were unable to adjust for their effects on osteoporosis risk in our multiple regression models. Fourth, our dataset did not contain information on the type and duration of diabetes. Nevertheless, we can reasonably infer that the majority of cases were likely Type II diabetes, given its considerably higher prevalence compared to Type I diabetes. This inference is supported by a 2019 study, which reported that the standardized prevalence of Type II diabetes in women was 9% across all ages, and over 30% in those aged 60–79 years in 2014, compared to a mere 0.05% prevalence of Type I diabetes in women during the same period [85]. Therefore, we anticipate that the proportion of Type I diabetes in our study sample is relatively small and unlikely to impact our conclusions significantly. Fifth, information on medication usage was not available. It is known that medications for hypertension and diabetes can influence bone status. Some medications can increase fracture risk, while others may increase BMD [86]. Sixth, while we have data on areal BMD via DXA, bone microarchitecture and extensive laboratory data, including hormone profiles, bone markers, and vitamin D levels, were unavailable from the health examination records. Previous research has shown that postmenopausal women with type 2 diabetes had significantly lower BMD, serum osteocalcin, and osteopontin levels, which could be vital in osteoporosis screening for patients with diabetes [87]. A cohort study of patients with diabetes found that volumetric BMD measured by quantitative computed tomography was more reliable for diagnosing osteoporosis and assessing fracture risk compared with areal BMD measured by DXA [88]. Future research should incorporate more diverse measurements such as trabecular bone score and glycated hemoglobin HbA1c.

Despite the aforementioned limitations, this study possesses several strengths contributing to its scientific value. One of the key strengths is the large sample size. Moreover, the simultaneous consideration of various combinations of diabetes, anemia, and renal function in examining the association between fasting blood glucose and osteoporosis risk represents a comprehensive approach rarely seen in previous research. This detailed analysis allows for a more thorough understanding of how these co-existing conditions might influence the risk of osteoporosis, offering valuable insights for clinical practice and future research. Furthermore, the interplay between osteoporosis and the trio of diabetes, anemia, and chronic renal disease is intricate and multifaceted, involving various physiological pathways and mechanisms, as illustrated in Fig. 2. These conditions may interact and potentially worsen each other’s progression and complications. This complexity highlights the need for prospective and animal studies to confirm the associations and further investigate the underlying mechanisms.

## Conclusions

The findings from this retrospective medical review study showed that fasting blood glucose levels were independently and significantly associated with an increased risk of osteoporosis in women with coexistent conditions of diabetes, anemia, and chronic kidney disease beyond stage II. The results highlight the importance of diligent monitoring of both blood glucose and bone mineral density in these women, coupled with the initiation of early interventions. Maintaining long-term glycemic control is important to prevent diabetic complications, such as chronic kidney disease and anemia. Our findings offer further evidence of the benefits of stringent management in lowering osteoporosis risk. Moreover, the complexity of metabolic and renal dysfunctions in increasing osteoporosis risk necessitates a multidisciplinary approach. This should encompass nutritional status assessments, rigorous control of inflammation, maintaining hormonal balance, and meticulous medication management, all aimed at lessening the compounded risks associated with osteoporosis in patients with these complex health challenges.

## Data Availability

The data used to support the findings of this study are available from the corresponding authors upon request.

## Abbreviations

ALP: Alkaline phosphatase
BMD: Bone mineral density
CI: Confidence interval
DXA: Dual-energy X-ray absorptiometry
eGFR: Estimated glomerular filtration rate
HDL-C: High-density lipoprotein cholesterol
LDL-C: Low-density lipoprotein cholesterol
OR: Odds ratio
